# Methods for the Evaluation of Industrial Mechanical Pretreatments before Anaerobic Digesters

**DOI:** 10.3390/molecules25040860

**Published:** 2020-02-15

**Authors:** Helen Coarita Fernandez, Diana Amaya Ramirez, Ruben Teixeira Franco, Pierre Buffière, Rémy Bayard

**Affiliations:** DEEP Laboratory, Univ. Lyon, INSA Lyon, EA7429, F-69621 Villeurbanne, CEDEX, France; helen.coarita@insa-lyon.fr (H.C.F.); diana.amaya-ramirez@etu.univ-lyon1.fr (D.A.R.); ruben.teixeirafranco@insa-lyon.fr (R.T.F.); remy.bayard@insa-lyon.fr (R.B.)

**Keywords:** anaerobic digestion, mechanical pretreatments, agricultural wastes, rheology, physical properties

## Abstract

Different methods were tested to evaluate the performance of a pretreatment before anaerobic digestion. Besides conventional biochemical parameters, such as the biochemical methane potential (BMP), the methane production rate, or the extent of solubilization of organic compounds, methods for physical characterization were also developed in the present work. Criteria, such as the particle size distribution, the water retention capacity, and the rheological properties, were thus measured. These methods were tested on samples taken in two full-scale digesters operating with cattle manure as a substrate and using hammer mills. The comparison of samples taken before and after the pretreatment unit showed no significant improvement in the methane potential. However, the methane production rate increased by 15% and 26% for the two hammer mills, respectively. A relevant improvement of the rheological properties was also observed. This feature is likely correlated with the average reduction in particle size during the pretreatment operation, but these results needs confirmation in a wider range of systems.

## 1. Introduction

In the field of anaerobic digestion, a vast number of reviews have been published in the last twenty years about substrate conditioning and pretreatments of organic biowaste [1,2,3], or more specifically, on different products such as municipal wastewater sludge [4], municipal organic solid wastes [5,6,7,8], lignocellulosic biomass [9,10,11,12], and agricultural wastes [13]. Even with an extensive and diverse research literature about different substrates, pretreatments, and the biomass recovery sector, research on mechanical pretreatments is still, to our knowledge, limited [14]. However, mechanical pretreatments are the most commonly used devices at full-scale [15,16]. Many functions and objectives are attributed to mechanical (physical) pretreatments. One of the most important objectives is to upgrade the digester feeding conditions in order to avoid floating layers [1] and reduce the size of materials and, consequently, to improve mixing, heat, and mass transfer [17]. Biomass pretreatment also extends to the types of feedstocks usable in anaerobic digestion The reduction in particle size leads to an increment in the accessible surface area and may increase methane production, either by increasing the methane yield or by increasing the methane production rate [18]. Nevertheless, physical pretreatments require considerable energy, ranging from 1 to 50 kWh per ton of fresh matter [3,18,19].

Different techniques are cited in the bibliography in order to perform substrate comminution, grinding or milling. Instruments, such as ball mills, knife mills, vibratory mills, hammer mills, and extruders, are used. Kratky and Jirout [3] concluded that the adequate device will mainly depend on the moisture content of the substrate. Knife and hammer mills were cited as the most suitable techniques for dry matter comminution, while extruders are more suitable for wet matter. In addition, they point out that particle size reduction will depend on the feeding system and the equipment operation (e.g., rotational rate). However, studies on mechanical devices are principally focused on energy requirements [3,20], and generally their functions and integral performance description are not explained. Thus, the performances of industrial mechanical pretreatments are mostly found in commercial booklets. In this context, knife mills are supposed to comminute and shear the biomass. Hammer mills break up the matter through a crippling effect which is a more advanced treatment. On the other hand, discs mills fragment and compress the biomass; string mills pull fibers of biomass reducing their size and cylindric mills dilacerate and decompact the biomass. There are also grinding pumps, pulpers, presses, and extruders, and their main functions are to reduce particle size and improve the fluidity. However, some mechanical pretreatments have been evaluated at full-scale such as cross-flow grinders [14,21], ball mills [22], and knife mills [23]. In these studies, the recurrent parameters investigated were the methane production yield (BMP) and the methane production rate, before and after pretreatment. Some authors also evaluated the potential release of inhibiting compounds during pretreatments through BMP tests and continuous experiments [14,15,24,25,26]. Tsapekos et al. [27] proposed physicochemical analysis, such as electrical conductivity tests, soluble chemical oxygen demand tests, and enzymatic hydrolysis on lignocellulosic biomass, as part of a pretreatment evaluation in order to find a correlation with the methane yield. Other methods, such as the measurement of the accessible surface area and of the water retention capacity, were suggested to evaluate the accessibility as an indicator of pretreatment efficiency [28]. The results obtained were interesting but not conclusive. By their side, Cai et al. [29] summarized some physicochemical characterization methods on lignocellulosic biomass such as particle size, grindability, density, flowability, and moisture adsorption, among others. Those analytical methods were pointed out as relevant for the process performance. Indeed, evaluation methods of a pretreatment must be in accordance with its main objectives. In summary, kinetics, biochemical, and solubilization parameters are the most common indicators to evaluate the pretreatment performance. However, physical parameters evaluations are also important, and their evaluation could be innovative to assess the benefits of the process in terms of energy consumption (lower mixing) and maintenance cost.

Parameters, such as size particle distribution, water retention capacity (WRC) and fluidity, are modified by a physical pretreatment. Water retention capacity is defined as the permanently bound amount of water attached to substrate when an external suction force is applied [30]. It is related with some properties of the substrate’s nature, such as porosity, and it can be an indicator of accessibility improvement [31]. In addition, water availability is important to make a substrate more flowable [32]. A substrate’s rheological properties play an important role in reactor mixing; among other parameters, homogeneity, mixing energy, and heat and mass transfer depend on these properties [33,34]. Many studies have demonstrated that rheological properties are related to a substrate’s physico-chemical properties, total solids (TS) content, particle size, and temperature [35]. Further research on the influence of mechanical pretreatments at the industrial scale on the rheological behavior of untreated and treated substrates with high TS and long-sized fiber contents is important in order to evaluate their performance. Mönch-Tegeder et al. [36] reported that smaller particles resulted in an improvement of the flow behavior. Indeed, energy efficiency in anaerobic digestion from unit operations is deeply associated to flow rheology characteristics. The pattern and the specific features, such as agitators and pumps, depend on raw and digested manure’s rheological behavior [37]. As an example, mixing in the reactor is an important energy factor to be considered, because it avoids significant gradients in the concentration of nutrients, inhibitor substances formation, suspended biomass and solids, temperature or pH. Besides, it limits the sedimentation of heavy solids, the formation of floating foams and scum as well as promotes the transfer of biogas [36,37,38].

In short, contrary to chemical, thermal or enzymatic pretreatments, mechanical pretreatments before anaerobic digestion have received very little attention by the research community. Despite this, it is clear that mechanical treatments affect the biochemical properties, soluble fractions, particle size, and rheological behavior of a given substrate.

The main novelty of the present work is the proposal of a comprehensive method to evaluate mechanical pretreatments at full-scale. In addition to biochemical parameters, physical parameters, such as size reduction, rheological properties, and water retention capacity, were evaluated. This method was tested on two anaerobic digesters using two different hammer mills as pretreatments.

## 2. Results and Discussion

### 2.1. Characterization of Incoming Products

Incoming products of both sites were relatively similar (Table 1). The TS (%TW) was 19.6% and 23.9% for sites I and II, respectively. For versus VS (%TS), a higher amount was found for Site I in relation to Site II. The pH values were similar for both sites and were typical of these kinds of substrates [14,24]. The COD and TKN concentrations were similar for Sites I and II as well. The ammonia nitrogen concentration was found to be higher in Site I, but the difference for this parameter is not uncommon [39].

### 2.2. Effect of Mechanical Pretreatments on Biochemical Characteristics

**Figure 1:**
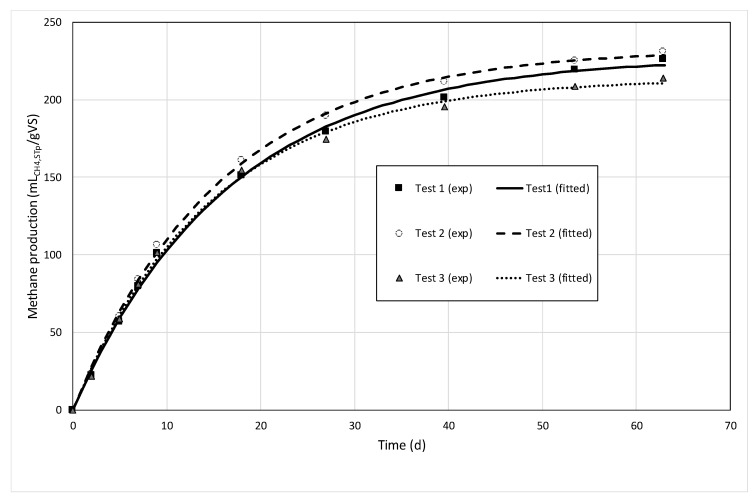
Methane production versus time for the biochemical methane potential (BMP) tests performed on Site II, after pretreatments (triplicate tests). The fitted curves represent the fitted first-order kinetic model.

An example of the methane production is shown in Figure 1. The methane production followed a first-order kinetics for all samples. The biochemical methane potential of raw cattle manure was much higher in Site I than in Site II (275 and 199 NL/gVS, respectively). This indicates that the manure of Site II is probably stored longer before use (which is confirmed by the elevated amount of ammonia nitrogen). However, the single factor ANOVA test found no significant differences between the BMP values of untreated and pretreated samples for both sites. This shows that mechanical pretreatments had no significant impact on the intrinsic biodegradability of the substrates. Divergent results can be found in the literature. Indeed, previous works made at full-scale found an enhancement of methane production by 10% with horse manure as a substrate [21]. Likewise, an enhancement of 26% on methane production was also reported with a cross-flow grinder pretreatment for horse manure [14]. Other available data reports an enhancement between 15% and 45% using different mechanical devices with manure fibers as substrate at laboratory-scale [15]. Dahunsi [40] found an average improvement of 22% of the BMP on different types of lignocellulosic substrates. Overall, the substrate biodegradability with mechanical pretreatments application depends on different factors such as the substrate nature and mechanical devices and their specificities.

The first-order kinetic constant *k* measured during the BMP tests was considered an indicator of the methane production rate. This parameter was improved by the mechanical pretreatments. Single factor ANOVA tests found significant differences in *k* for the untreated and treated samples for both sites. An improvement of 15% and 27% with the pretreatment application was found in Site I and Site II, respectively. Published results with manure as a substrate and mechanical pretreatments at full-scale are scarce and difficult to compare. Mönch-Tegeder et al. [21] pointed out a small effect on degradation kinetics with a cross-flow grinder device application at full-scale. These results agreed with Moset et al. [41], who found similar *k* values between excoriating and chopping grass pretreatment, unfortunately untreated samples were not evaluated. For their part, Herrmann et al. [25] described an enhancement of approximately 18% on the methane production rate with a chopping pretreatment of lignocellulosic feedstocks at farm-scale. In contrast, a remarkable enhancement (approximately 43%) was found by Dumas et al. [23] at the laboratory-scale with wheat straw mechanical pretreatment. In short, the methane production rate not only depends on the size reduction but also on other parameters such as the fiber structure and the chemical characteristics [42,43].

Probably, laboratory-scale devices provide higher energy levels than full-scale ones which results in a higher extent of biomass degradation and, consequently, a higher impact on both the BMP and on the methane production rate. For instance, a 500 W laboratory-scale blender treating 1 kg of manure in 5 min delivers around 42 kWh/t of fresh manure which is four times higher than the energy consumed by the hammer mills (10 kWh/t). In the work by Dahunsi, the energy required for the pretreatment comprised between 300 and 350 kWh/t (of total solids), while in our case, at real scale, the energy was lower (10 kWh/t of fresh matter at 20% TS was 50 kWh/t of TS).

Volatile fatty acids (VFAs) content increased with pretreatment for Site I, from 1.1 to 24.7 mg/gVS in contrast to Site II which presented almost no difference. However, this increase was less than 3% of the initial volatile solid content. No losses of VFA were reported through warming by mechanical pretreatments of lignocellulosic silage and manure [14,22]. An increment of VFA concentration could be attributed to a better VFA solubilization [22,26]. In addition, the substrate nature’s, such as the initial proteins contents, and its fermentation may affect VFA concentration [26]. In all samples, no soluble sugars were detected.

### 2.3. Effect of Mechanical Pretreatments on Physical Characteristics

#### 2.3.1. Size Reduction

**Figure 2:**
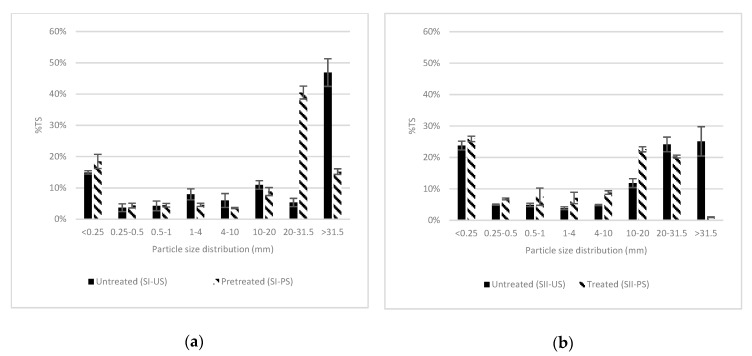
Particle size reduction before and after hammer milling pretreatment: (**a**) Site I; (**b**) Site II.

Figure 2 shows the particle size distributions of the untreated and treated samples for Site I and Site II. For Site I, almost 50% of the total solids were composed of fibers larger than 31.5 mm. The second largest proportion was fibers shorter than 0.25 mm (approximately 15%) followed by fibers between 20 and 10 mm (approximately 11%); the other measured proportions (20–31.5 mm, 4–10 mm, 1–4 mm, 0.5–1 mm, 0.25–0.5 mm) were less than 10%. For Site II, the same proportions (approximately 25%) were found on samples higher than 31.5 mm, between 31.5 and 20 mm, and lower than 0.25 mm. Fibers between 10 and 20 mm accounted for more than 12% of the total amount of solids. Thus, initial differences may be found between the untreated Site I and Site II samples’ fiber sizes. Site I presented a bigger fraction of larger fibers than Site II. Nevertheless, similarities were also found between the two evaluated samples; the biggest proportion for each sample were fibers longer than 31.5 mm and shorter than 0.25 mm.

Size reduction behavior after mechanical pretreatment was relatively similar for both sites. Site I sample fibers >31.5 mm were mainly reduced to 20–31.5 mm in size. Indeed, the decrease of longest measured fibers was approximately 22%, and the amount of fibers between 20 and 31.5 mm in length increased by approximately 35%. For Site II, the fibers >20 mm were mainly reduced to 10–20 mm in size. Initially, fibers longer than 31.5 mm were reduced by more than 20%. Then, fibers between 20 and 31.5 mm were reduced, and the amount of fibers between 10 and 20 mm increased by 10%. The proportion of fibers sized 4–10 mm, 1–4 mm, 0.5–1 mm, 0.25–0.5 mm, and lower than 0.25 mm were in all the cases reduced to a smaller size. In short, the reduction in particle size was produced mainly on longer fibers and a slight increase on the smallest particles was found with mechanical pretreatments. Sun and Cheng [44] pointed out a reduction of lignocellulosic substrate particle size between 10 and 30 mm with a chipping pretreatment and 0.2–2 mm with a grinding or milling operation. Indeed, particle size reduction depends on many factors related to the mechanical device and its operation in addition to the substrate’s properties such as humidity and initial particle size [3,10,25,45]. Furthermore, size reduction is commonly reported as a factor explaining higher methane production yields and methane production rates due to the increase in the accessible surface area [46,47].

#### 2.3.2. Water Retention Capacity

**Figure 3:**
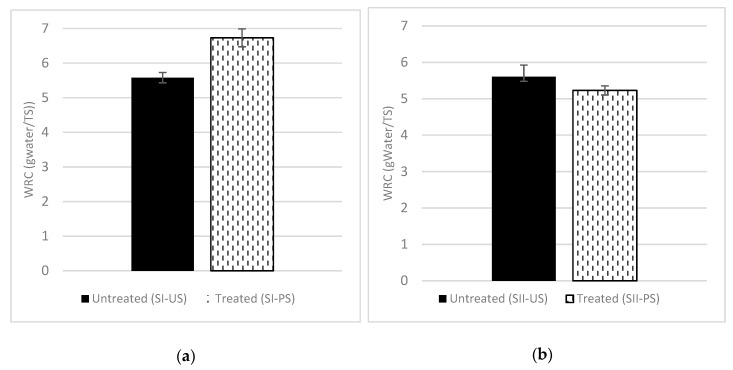
Water retention capacity of untreated and treated samples: (**a**) Site I; (**b**) Site II.

The WRC results were relatively similar for Site I and Site II (Figure 3). On the one hand, the WRC increased with the pretreatment for Site I from 5.6 ± 0.2 g water/TS to 6.7 ± 0.3 g water/TS (around +19%). On the other hand, for Site II, no significant changes were obtained (WRC of 5.6 ± 0.3 g water/TS to 5.2 ± 0.1 g water/TS). The ANOVA tests found significant differences for WRC values for Site I. These results suggest that the pretreatment improved the water retention capacity as consequence of particle size reduction [32]. In contrast, an excess of particle size reduction could lead to a decrease of WRC. Indeed, water behavior was not changed for Site II, and it could probably be due to the fact of its higher amount of particles smaller than 0.25 mm and contents with shorter fibers than Site I. Dumas et al. [23] noted that water behavior does not change considerably if the particle size becomes lower. Other authors [48,49] have suggested improvements in terms of biodegradability when the WRC increases, because it may favor nutrients and substrate dissolution and diffusion. No relation was found between biodegradability and WRC in our case.

#### 2.3.3. Rheological Properties

Yield stress and apparent viscosity decreased when mechanical pretreatments were applied at similar TS contents of untreated and treated samples (Table 2). A reduction in the yield stress of 63% and 18% was observed for Sites I and II, respectively. SI-US apparent viscosity in Site I could not be measured due to the equipment limitations. In Site II, a reduction of 85% of the apparent viscosity was recorded with the pretreatment. The difference among the results of each site can be explained by the fact that the rheological properties are also linked to particle size distribution [50]. Particle sizes > 31.5 mm (Figure 2) were approximately 20% higher at Site I than Site II before pretreatment. The yield stress values were three times higher for Site I than Site II at similar TS. According to Tian et al. [38], the required energy for appropriate mixing of the digester may be reduced by 9.2% with a particle size reduction from 20 to 80 mesh (with corn stover as a substrate). In addition, the mixing energy is (in first approximation) directly proportional to the apparent viscosity of the digester content [51]. Thus, it was found that higher contents of long particle size increased the yield stress and viscosity values, and mechanical device application had a positive impact on rheological properties. A similar trend was reported with tests made with agricultural wastes as a substrate by different authors [14,38,44]. In addition, flow and viscosity behavior did not necessarily correspond at similar biomass TS values according to published data [33,35].

As mentioned previously, the reduction in the yield stress and viscosity values could mean an important reduction of energy consumption in terms of pumping and mixing at full-scale sites [36,52]. Moreover, the relationship between high solids concentration and different particle sizes should be investigated more in detail for a better comprehension of rheological properties, biodegradability, hydrophilicity, and mechanical pretreatments influence.

## 3. Materials and Methods

### 3.1. Biomass Sampling and Handling

Two full-scale plants with mechanical pretreatment operations were selected for this study. In both units, cattle manure was the main feedstock.

Site I: The mechanical pretreatment was a mobile *hammer mill*. Samples were collected before and after the hammer mill device and just before the anaerobic digestion tank. Untreated and treated samples were stored at 4 °C and analyzed within a few days. At this site, a mesophilic digester is operated. Mechanical pretreatment is used to improve the feeding system and to avoid digester difficulties such as clogging.

Site II: A fixed (in-line) *hammer mill* was evaluated. Samples were collected before and after the mechanical pretreatment operation. This site operates with a mesophilic digester and produces heat and electricity from biogas. This mechanical operation is mainly used to reduce the fiber size. At each site, 50 kg of untreated and 50 kg of treated manure was sampled. The samples were transported and stored in a 4 °C cold chamber and processed less than 48 h after storage for further analysis. 

### 3.2. Biochemical Characterization

The biochemical characterization of the samples followed the procedure suggested by Teixeira Franco et al. [53]. A fractionation of the sample was made through a leaching test with water. The leaching test was performed with 10:1 water/dry matter proportion over 2 h under a constant bottle rotation. Subsequently, the sample was centrifuged (20 min at 5000× *g*). The liquid fraction was filtrated (1.2 µm), while the particulate fraction was dried at 70 °C and ground to 1 mm length. The measurements carried out on the biomass samples are described below.

Analyses on the raw (untreated) sample: parameters as total solids (TS); volatile solids (VS); and biochemical methane potential (BMP) test assays were carried out. The samples were dried at 105 °C with a drying oven in order to measure TS; afterwards, samples were burned at 550 °C then versus were calculated.

Analyses on the liquid fraction: liquid fraction was used to measure pH, dissolved chemical organic demand (COD); ammonia nitrogen (NH_3_-N); total Kjeldahl nitrogen (TKN); volatile fatty acids (VFAs); BMP; TS; and VS. The pH was measured with a consort C3020 device with SP10B. Lactic and formic acids were analyzed with a high-performance liquid chromatography (LC Module 1 plus, Waters) equipped with a Supelcogel™ C-610 H column (300 mm × 7.8 mm, Sigma–Aldrich), with both refractive index (RID) and UV detectors and operating with H_3_PO_4_ 0.1%v/v as solvent (flow rate of 0.5 mL/min). Acetic, propionic, butyric, valeric, and caproic acids contents were analyzed by gas chromatography (Shimadzu Corp.) with an HP-FFAP fused silica capillary column (30 m × 0.25 mm, Agilent Technologies), a flame ionization detector, and H_2_ as the carrier gas. The sum of lactic, formic, acetic, propionic, butyric, valeric, and caproic acids was considered as the total VFA. The TKN and NH_3_-N were determined according to the NF EN 25,663 standard procedures.

Analyses on the particulate fraction: Parameters, such as TKN, COD, total solids (TS) and volatile solids (VS) were determined.

The BMP tests were performed according the guidelines given by Holliger et al. [54]. Batch assays were prepared in 1 L glass bottles in a 35 °C temperate room. The inoculum used was a digested sludge from a wastewater treatment plant (La Feyssine, Lyon, France). The total versus content was 20 g/L (sample + inoculum), and the substrate to inoculum ratio was 0.5 on VS basis. A mineral solution was added (as recommended by the ISO 117734:1995 standard). The mineral solution contained essential elements in order to give optimal conditions to the microbial growth and act as a buffer solution. Then glass bottles were purged with 80/20%v/v N_2_/CO_2_ and finally incubated. Control assays without substrate were performed as well in order assess the background methane production from inoculum. The gas production was followed by manometric measurements (Digitron precision manometer). The biogas was vented when the pressure went above 1200 hPa. The biogas composition was analyzed with an Agilent 300 micro gas chromatography with a thermal conductivity detector (GC-TCD). Argon and helium were used as carrier gases and Molsieve 5 A (14 m length; pore size: 5 Å) and PoraPlot (10 m length; 0.320 mm ID) columns as stationary phases. Each sample was tested in triplicate.

The background methane production (from inoculum) was retrieved, and the methane volume produced was normalized and expressed in ml standard conditions for temperature and pressure (0 °C, 1 atm) per gram of volatile solid. From the methane production, a first-order model was fitted to the data in order to get further information from the experiments:

(1) $$V_{CH4}\left(t\right)=BMP_{max}\left(1-e^{-kt}\right)$$

- the final (or ultimate) maximal biochemical methane potential, *BMP_max_*;

- the first-order kinetic parameter *k* that can be compared prior and after the mechanical pretreatment as an indicator of methane production rate improvement.

### 3.3. Physical Characterization

#### 3.3.1. Size Reduction Evaluation

Particle size distribution was measured to evaluate the effects of the mechanical devices on particle size. Different procedures are proposed in the literature. Herrman et al. [25] used a quantification of ensiled feedstock by image analyses with 10 to 20 g of sample. Samples were weighted, scanned, and evaluated with an image analyzer software. In contrast, Mönch-Tegeder et al. [21] performed a wet sieving for horse manure and ensiled feedstocks at a determined size and then evaluated them with an image analyzer software. Lindner et al. [22] also used a wet sieving with a vibrating sieve shaker in order to verify the size reduction of digestate composed of cattle manure and silage. Samples were soaked in distilled water, and then samples were sieved (10 min), weighted, and dried. On the other hand, Tsapekos et al. [27] used a visual method to analyze ensiled meadow grass particle distribution. Indeed, these procedures depend on the substrate conditions and research aim for evaluating different particle sizes. Sieving is often used to measure particle size distribution due to the fact of their ample range. Nevertheless, some caution must be taken to prevent from clogging, which would result lower amounts of small particles [55].

The procedure carried out in this work was also a wet sieving method with a vibrating sieve shaker (Retsch AS 200 basic) with water recirculation. The sieve openings were 0.25 mm, 0.5 mm, 1.0 mm, 4.0 mm, 10.0 mm, 20.0 mm, and 31.5 mm. First, 50–80 g of samples was weighted, disaggregated, and carefully introduced into the upmost sieve (31.5 mm). The amount of sample was chosen in order to prevent any clogging of the sieves. Then, samples were sieved with 1 L of recirculated demineralized water for 15 min. A flow of 938 mL/min until a 40 amplitude vibration was performed. Water was retrieved, and it was reinjected for 5 min of sieving. All fractions were recovered with aluminum paper (weighted previously) and dried over 24 h at 105 °C. The size distributions were calculated in %TS, and their proportion corresponded to the total dried weight. For each sample, this operation was done in triplicate.

#### 3.3.2. Rheological Properties

According to different authors, fermenting agricultural biomasses has been shown to have a rheofluidifying behavior characteristic of non-Newtonian fluids [33,35,36,37,56] where viscosity decreases non-linearly under a shear stress. Conventional methods for measuring rheological properties are mostly performed with rotational or tubular rheometers in order to build a rheogram and, consequently, to identify the behavior fluid model [56]. These measurements relate shear stress in Pascal (Pa) and shear rate (s^−1^) depending on the force applied to the fluid [52]. In addition, other characteristics may influence the rheological properties as the temperature, the TS content, the particle size, and the presence of dispersed gas bubbles [36]. Indeed, rotary and tubular devices are not adapted for long and heterogeneous straw fibers present in cattle manure. Their size and capacity to measure only small and homogeneous quantities of sample are not representative at full-scale. By consequence, these measurements with heterogeneous substrate are difficult to investigate. Thus, information about measurement methods of rheological properties on heterogeneous samples with important fiber sizes at the industrial scale is still limited [42,44].

A few studies in this field were found in the literature, one of them by Mönch-Tegeder et al. [36], who developed an in-line process viscometer at full-scale in order to determine the rheological properties of untreated and pretreated mixed substrates with grass silage, maize silage, and solid and liquid manure. The viscosity increased with the fibrous material, and the substrate’s mechanical disintegration positively influenced the rheological properties; however, an important effect of higher TS was pointed out.

Moreover, Ruys [50] proposed a dedicated rheometer of dimensions larger than commercial ones and adapted to heterogeneous substrates before anaerobic digestion at industrial scale. However, this kind of prototype is not commercially available; moreover, it is complex to operate at industrial scale. In this context, Garcia-Bernet et al. [48] developed a fast method to measure the yield stress for anaerobically digested solid waste with a slump test. It involves a cylindrical shape PVC chamber with a 10 cm diameter and 18 cm height; this chamber is filled with the sample, then it is quickly lifted vertically to allow the medium to collapse, and the difference between the initial and final heights is termed the “slump”.

We tested two devices commonly used for fresh concrete characterization. These devices are able to measure the yield stress [57] and the apparent viscosity [58] using a slump test and a V funnel test, respectively. Yield stress value represents the minimum stress to cause the fluid flow, and its measurement is essential for understanding flow properties [57]. Apparent viscosity represents a fluid’s property to resist forces causing it to flow; thus, a flow’s velocity is controlled by these internal resistances of the fluid [52]. These internal variations become a key factor during the application of a force on the fluid for its correct handling and management [59].

The slump and V funnel tests were carried out according to the PR NF EN 12350-2 and PR NF EN 12350-9 standards used for fresh concrete, respectively, and the TS content of the samples was standardized to 11%–12% approximately. The experiments were performed at 20 ℃. The slump test was carried out with an Abram cone, and the results were evaluated with a cylindrical analytical model as Pashias et al. [57] suggested, where the slump height is related to yield stress. Dimensionless height can be expressed by Equation (1), where dimensionless slump height is defined as $s^{\prime }=s/H$, dimensionless yield stress is defined by $\tau_{y}^{\prime }=\tau_{y}/\rho gH$, and H is the cone’s height, H = 30 cm.

(2) $$s^{\prime }=1-2\tau_{y}^{\prime }\left[1-Ln\left(\tau_{y}^{\prime }\right)\right]$$

**Figure 4:**
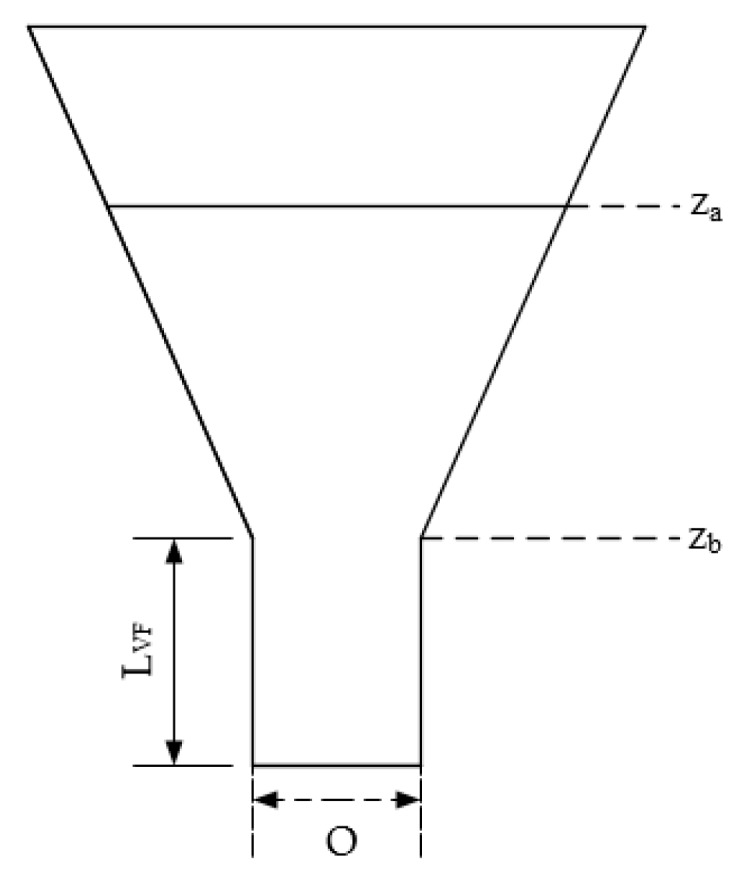
Schematic view of the V Funnel adapted from Mokéddem (2012) [47].

Apparent viscosity $\eta_{app}$ was calculated with Equations (3)–(5) described by Mokéddem [58], where *dP* is the difference in pressure between the V funnel input and output, *t_VF_* is the flow time through the V funnel in seconds (s), *V_VF_* is the V funnel volume (10 L), and *L_VF_*, *O*, *z_a_*, and *z_b_* are the V funnel dimensions as shown in Figure 4.

(3) $$dP_{mean}=\frac{dP_{max}}{2}$$

(4) $$dP_{max}=\rho g\left(z_{b}-z_{a}\right)$$

(5) $$\eta_{app}=\frac{dP_{mean}t_{VF}O^{4}}{24L_{VF}V_{VF}}$$

#### 3.3.3. Water Retention Capacity (WRC)

There are various methods to measure the water retention capacity of a given sample; and most of them have been used in the food industry [30,60,61] and for lignocellulosic biomass [23,32]. A centrifugation of samples with distilled water was applied in most of the cases. Sanchez et al. [32] assembled a vacuum system with a Buchner funnel, filter paper, and a Kitasato flask. Regarding the evaluated substrate, a procedure considering a higher mass amount was privileged. Similarly, we used a column with a filter cloth system and a vacuum pump inserted into an Erlenmeyer flask, and 250–500 g of raw sample and distilled water were inserted into the column. Water was carefully added in order to have total contact with the sample at ambient temperature for 2 h. Water was pulled out via gravity by opening the tap and under slight vacuum for 120 s. The measurement was calculated as suggested by Raghavendra et al. [61]; the water quantity was retained over the TS substrate. Analyses were performed in triplicate for all samples.

## 4. Conclusions

The present work developed a comprehensive methodology to investigate the effect of mechanical pretreatments in the context of agricultural waste anaerobic digestion including biochemical and physical characteristics. Conventional methods included solid/liquid separation, BMP measurement, and solubilization characteristics. Physical methods included particle size distribution, water retention capacity, and rheological characteristics. Our methodology was tested on sites with two different hammer mills.

The results showed no improvement in the methane yield (BMP) before and after the pretreatments. However, the methane production rate increased significantly (+15% and +27% for Sites I and II, respectively). These results were quite in line with the increase in water retention capacity, indicating that the organic matter was somewhat more easily accessible to hydrolysis.

Rheological properties were assessed by rapid tests (the Abram cone and the V funnel) that can be easily used on site. Pretreatments improved both the yield stress (−18% and −63% for Sites I and II, respectively) and the apparent viscosity (−85% for Site II). The average particle size reduction played a significant role in these improvements. Since rheological parameters are directly linked with the energy required for digester mixing, these tests are very promising tools for further evaluation of pretreatments at full scale.

## Figures and Tables

**Table 1:** Physical and biochemical characteristics of untreated and pretreated samples.

		Site I		Site II	
**Parameter**	**Unit**	**SI-US** ^1^	**SI-PS ^2^**	**SII-US ^3^**	**SII-PS ^4^**
TS	%(TW *)	19.6 ± 1.0	18.8 ± 0.5	23.9 ± 1.1	25.8 ± 0.4
VS	% (TS)	88.4 ± 0.9	87.1 ± 0.1	83.0 ± 3.4	75.6 ± 2
pH	-	8.23	8.04	8.28	8.27
COD	mg/g VS_RS **_	1305	1317	1215	1219
TKN	mg/g VS_RS **_	27.0	25.7	21.0	23.5
*NH_4_-N*	mg/g VS_RS **_	6.2	3.9	1.7	1.5
*VFA*	mg/gVS_RS **_	1.7	24.7	4.3	2.7
BMP	mL/gVS_RS **_	275 ± 7	269 ± 10	199 ± 15	224 ± 7
*k*	j^−1^	0.066 ± 0.005	0.077 ± 0.004	0.055 ± 0.004	0.070 ± 0.004

^1^ SI-US: untreated samples; ^2^ SI-PS: pretreated samples; ^3^ SII-US: untreated samples; ^4^ SII-PS: pretreated samples. * TW: total weight; ** RS: raw sample.

**Table 2:** Rheological properties for untreated and treated samples.

		Site I		Site II	
Parameter	Unit	Untreated *	Pretreated *	Untreated **	Pretreated **
*τ_y_*	Pa	755.3 ± 18.4	281.0± 4.2	250.7 ± 6.6 **	205.0 ± 16.1
*η_app_*	Pa.s	ND ***	28.6 ± 3.9	38.7 ± 2.1	5.8 ± 0.2

* TS = 12%; ** TS = 11%; *** ND = non-determined.
